# Symbiotic Plant-Bacterial Endospheric Interactions

**DOI:** 10.3390/microorganisms6020028

**Published:** 2018-03-22

**Authors:** Sharon Lafferty Doty

**Affiliations:** School of Environmental and Forest Sciences, University of Washington, Seattle, WA 98195-2100, USA; sldoty@uw.edu

While plant-microbe symbioses involving root nodules (Rhizobia and Frankia) or the root-soil interface (rhizosphere) have been well studied, the intimate interaction of microbial endophytes with the plant host is a relatively new field of research. Nutrient acquisition, phytohormone production and modulation, antimicrobial production, and native and xenobiotic chemical detoxification are all ways in which the plant microbiome can impact plant health. These interactions may be essential to how plants in their native environments survive and thrive, especially in challenging environments. Knowledge of the beneficial plant-microbiome interactions also has obvious implications in agriculture, forestry, and bioenergy for improved environmental sustainability.

This special issue covers the spectrum of the plant-bacterial endophyte interactions, from the transmission of the microorganisms and subsequent colonization of the host plant, to analysis of the microbiome and constructed communities, to the impact on the host plant for growth promotion and tolerance to both abiotic and biotic stresses. The article by Frank et al. provides a comprehensive review of the ways in which members of the plant microbiome can be transmitted [1]. While some bacterial endophytes can be vertically transmitted through seeds, the majority seem to come from the environment by air, water, or soil. Even the microbiome of seeds and pollen may actually be externally derived. Pollinators and sap-feeding insects may also be important in transmitting microbiota between plants. The article by Kandel et al. reviews how endophytes colonize within the plants, and provides new data on cross-species colonization between divergent hosts [2]. The review discusses the importance of endophytes, how plants may recruit endophytes from the environment, the process of attachment and entry into the host, niches within the plant, bacterial genes required for colonization, the colonization cycle, and methods of studying endophytic colonization. The review then uses the colonization of the monocot, maize, by endophytes of the eudicot, poplar trees, as an example to illustrate the different steps and outcomes of effective colonization. Moving from the analysis of the Venezualian rice microbiome to constructed communities and how they behave, the research article by Morronta-Barios et al. provides an example of how strains could be selected for plant growth promotion [3]. Using auxin production as the primary screen, the authors selected strains to more fully characterize in vitro, narrowing down to 10 strains to test as a constructed community in vivo. The authors provide interesting new data on the outcomes of the individual strains over time. The Special Issue also includes three articles on using endophytes for improving tolerance to abiotic and biotic stresses, one on drought tolerance and two on the antimicrobial activities of endophytes. The article by Cura et al. describes the successful use of the common endophyte inoculum from sugarcane to impart drought tolerance in maize [4]. Compared to the control plants, the inoculated maize had greater biomass, higher levels of carbon, nitrogen, and chlorophyll, decreased levels of the stress hormones, ABA and ethylene, and higher relative water content. In the inoculated plants, indicators of osmotic stress were at levels similar to the unstressed controls. Considering that drought stress results in yield losses of nearly 40% in maize, the ability of the standardized inoculum of *Azospirillum brasilense* and *Herbaspirillum seropediciae* to increase drought tolerance has important implications for agriculture. The review by Khan et al. describes the multiple mechanisms by which endophytic *Bacillus* species can combat the plant pathogenic fungus *Fusarium* [5]. This broad host range pathogen causes numerous agriculturally important diseases including damping off, root rot, wilting, head blight, and premature leaf drop. The authors provide specific examples of how the non-pathogenic endophytic *Bacillus* species can inhibit *Fusarium* through direct competition for nutrients, by inducing the plant’s systemic resistance system, and by producing antifungal compounds. It was noted that future directions of biocontrol research could explore consortia of multiple *Bacillus* species since these were especially effective at blocking the pathogen. The seventh article in the Special Issue describes the selection and successful use of endophytes to protect seedlings from *Fusarium* infection and enhance the growth of rice [6]. The authors found that three *Pseudomonas* spp. endophytes were effective at blocking the fungus in vitro in plate assays and also in vivo by protecting rice seedlings from fungal attack. Possible mechanisms were explored using microscopy to analyze for mycelial lysing, and MALDI-TOF and genomics approaches for potential antimicrobial synthesis. Overall, the papers in this Special Issue nicely illustrate how beneficial endophytes are acquired and the breadth of their impacts on plant growth and health.
